# Comparison of outcomes and cost-effectiveness of laparoscopic and open appendectomies in public health services

**DOI:** 10.1590/0100-6991e-20213010

**Published:** 2021-09-28

**Authors:** JOÃO HENRIQUE FONSECA DO NASCIMENTO, BENJAMIM MESSIAS DE SOUZA, SELTON CAVALCANTE TOMAZ, ADRIANO TITO SOUZA VIEIRA, BERNARDO FERNANDES CANEDO, ANDRÉ BOUZAS DE ANDRADE, ANDRÉ GUSMÃO-CUNHA

**Affiliations:** 1 - Universidade do Estado da Bahia (UNEB), Departamento de Ciências da Vida - Salvador - BA - Brasil

**Keywords:** Appendectomy, Laparoscopy, Cost-Benefit Analysis, Public Health, Apendicectomia, Laparoscopia, Análise Custo-Benefício, Saúde Pública

## Abstract

Acute appendicitis is the leading cause of abdominal emergency surgery worldwide and appendectomy continues to be the definitive treatment of choice. This cost-effectiveness analysis evaluates laparoscopic versus open appendectomies performed in public health services in the state of Bahia (Brazil). We conducted a retrospective observational study using the database from the Department of Informatics of the Unified Health System (DATASUS). Available data on appendectomies between 2008 and 2019 were included, and we evaluated the temporal trend of hospital admissions, procedure-related mortality rates, length of stay, and costs. Statistical analysis was performed using the R-software (R Foundation, v.4.0.3) and the BioEstat software (IMDS, v. 5.3), considering p<0.05 as significant. During 2008-2019, 53,024 appendectomies were performed in the public health services in Bahia, of which 94.9% were open surgeries. The open technique was associated with a higher mortality rate (4.9/1,000 procedures; p<0.05) and a higher risk of death (RR=4.5; p<0.05) compared to laparoscopy (1.1/1,000 procedures). Laparoscopic appendectomy (median of 2.7 days) had a shorter length of stay compared to laparotomy (median of 4.15 days) (p<0.05). There was no difference in the medians of costs nor hospital services, per procedure (p=0.08 and p=0.08, respectively). Laparoscopic professional median costs were higher by US$ 1.39 (p<0.05). Minimally invasive surgery for appendicitis is a safe and efficacious procedure in Brazilian public health care services, as it provides advantages over the open method (including lower procedure-related mortality rate and earlier discharges), and it did not imply higher expenses for public service budgets in the state of Bahia.

## RESUMO

Apendicite aguda é a principal causa de cirurgia abdominal de emergência no mundo e a apendicectomia continua sendo o tratamento definitivo de escolha. A presente investigação avalia desfechos e custos das apendicectomias laparoscópicas versus abertas realizadas em serviços públicos de saúde no estado da Bahia (Brasil). Realizou-se estudo observacional retrospectivo, utilizando a base de dados do DATASUS. Incluiu-se dados disponíveis sobre apendicectomias na Bahia entre 2008 e 2019, avaliando-se a tendência temporal de internações, taxas de mortalidade por procedimentos, tempo de permanência e custos. A análise estatística foi realizada no R-software (Fundação R, v.4.0.3) e no software BioEstat (IMDS, v.5.3), considerando p<0,05 significativo. Entre 2008 e 2019, realizou-se 53.024 apendicectomias no serviço público de saúde na Bahia, das quais 94,9% foram cirurgias abertas. A laparotomia foi associada à maior taxa de mortalidade (4,9/1.000 procedimentos; p<0,05) e maior risco de morte (RR=4,5; p<0,05) do que laparoscopia (1,1/1.000 procedimentos). Apendicectomia laparoscópica (mediana de 2,7 dias) obteve menor tempo de internamento do que cirurgia laparotômica (mediana de 4,15 dias) (p<0,05). Não houve diferença entre as medianas dos custos e nem dos serviços hospitalares por procedimento (p=0,08 e p=0,08, respectivamente). A mediana do custo de profissionais na laparoscopia foi significativamente mais elevada, em US$ 1,39 (p<0,05). A cirurgia minimamente invasiva para apendicite é um procedimento seguro e eficaz, proporcionando vantagens sobre a laparotomia (incluindo menor taxa de mortalidade e alta precoce), não implicando, por sua vez, em maiores despesas para cofres públicos no estado da Bahia.

## INTRODUCTION

Acute appendicitis is the leading cause of emergency abdominal operations worldwide^1^ ^,^ ^2^. The incidence is approximately 233/100,000 people, with an estimated lifetime risk of 8.6% among males, and 6.7% among females^2^ ^,^ ^3^. Affected patients are typically between the ages of 5 and 45, and the disease is highly prevalent in the second and third decades of life^4^. In Brazil, it is responsible for more than 100,000 hospital visits yearly^5^. Despite the heterogeneous patterns of clinical presentation, appendectomy is the definitive treatment of choice for acute appendicitis until the present moment^2^ ^,^ ^4^, and it is one of the most commonly non-elective procedures performed by general surgeons^6^. 

Regarding the surgical approach, Charles McBurney first described the open traditional laparotomic appendectomy by the “McBurney incision” in 1889, and it was considered the gold standard treatment for acute appendicitis for over a century^6^ ^,^ ^7^. The technique is based on the incision made in the lower right quadrant of the abdomen, with exposure of the appendix and part of the colon^6^ ^,^ ^8^. It was only in 1981 that the German gynecologist Kurt Semm introduced the minimally invasive technique - the laparoscopic appendectomy^7^ ^,^ ^9^. 

Although the laparoscopic approach has been performed for almost 40 years now, the discussion on the applicability of these technique in public health services remains current^6^ ^-^ ^11^ due to its costs. Despite the advantages associated with laparoscopy for appendicitis, such as reduced length of stay, early return to social and labor activities, lower incidence of wound infections, less postoperative analgesia medication, faster overall recovery, and better aesthetic results^6^ ^,^ ^8^ ^,^ ^9^, several retrospective studies, meta-analyses, and randomized trials have reported conflicting results^7^.

Therefore, given the significance of the topic and the need for data that substantiate the refinement of public policies in hospital administration, we conducted an epidemiological study on the appendectomies performed in public health services in the state of Bahia, comparing the traditional open technique to the laparoscopic approach, and performed temporal trend analyses of the number of operations, mortality rates and financial costs related to these procedures.

## METHODS

This population-based, retrospective and observational study, carried out with secondary data from a government database of hospital procedures, evaluated the applicability and the cost-effectiveness between open and laparoscopic appendectomies in the state of Bahia (Brazil). The Unified Health System Department of Informatics (Departamento de Informática do Sistema Único de Saúde - DATASUS) is a public available online data platform, managed by the Ministry of Health, along with the state health secretariats (available at http://datasus.saude.gov.br/). The data on procedures were collected through the Hospital Information Systems (Sistema de Informação Hospitalares - SIH) from DATASUS, which gathers most of the information regarding the number of hospital admissions, surgical interventions, financial costs, and patient outcomes. DATASUS platform defines hospital admission as patients remaining in hospital for more than 24 hours, thus single day-hospital approaches were not included in the analyses. All the data were stratified geographically by the place of residence of patients (state of Bahia). 

To perform this investigation, we analyzed the following variables: total number of hospital admissions per procedure, total number of procedure-related deaths and the procedure-related mortality rate, total number of days and the average length of stay, total costs and the mean cost per procedure, as well as costs pertaining hospital and professional services. Regarding the financial analyses, the Brazilian real (R$) was divided by 5.2 to convert into dollars (US$ dollar price in December 2020). All of the available data were retrieved using the SIH code number 04.0702.00.39 for open appendectomies and the SIH code number 04.0702.00.47 for laparoscopic appendectomies, from January 2008 to December 2019.

The normality of the variables was assessed by the Shapiro-Wilk test. In addition, the homogeneity of the compared group variances was assessed with the Levene’s Test for Equality of Variances. Descriptive statistics such as mean, standard deviation (±SD), median, interquartile range (IQR: Q1 - Q3), besides relative risk (RR) and confidence intervals (CI) were used to describe numbers and proportions of admissions, procedure-related deaths, costs and length of hospital stay. 

The Fisher’s exact test and Chi-squared test with Yates’ continuity correction were used to compare proportions between two groups. Depending on the normal or non-normal distribution of the variables, a Mann-Whitney U test and a Student’s T test for independent samples were also used when appropriate to compare differences between groups. In order to assess the data variation along time, the percentage was calculated between the years applying the following formula: [(next year - previous year)/previous year] ×100, to identify the stability, increase or decrease of the numbers. The adjusted r² values were obtained using linear regression to evaluate the variance of trends, considering a p<0.05 result as significant. The forecasted data for the year 2025 were achieved using the triple exponential smoothing model for time series (confidence interval of 95%).

Data management and statistical analysis were conducted using the Microsoft Office Excel 2019 software (Microsoft, Redmond, Washington, USA), the BioEstat software (Instituto de Desenvolvimento Sustentável Mamirauá, v. 5.3) and the R software (RStudio, Inc. - R foundation for statistical computing, v. 4.0.3), a free from charge data analysis software.

Approval of the Ethics Committee in Research is considered dispensable, since secondary data were obtained from the public domain and online database, without identification of patients, as stated by the National Council of Health (Conselho Nacional de Saúde - CNS) and the National Commission of Research Ethics (Comissão Nacional de Ética em Pesquisa - CONEP) in Brazil (available at http://conselho.saude.gov.br/web_comissoes/conep/index.html).

## RESULTS

From 2008 to 2019, a total of 53,024 appendectomies were performed in public health services in the state of Bahia. Most of the procedures - 94.9% (n=50,302) - were made by the open technique, with a mean of 4,192 ± 585 surgeries per year (Table 1). Laparoscopic approach was accountable for 2,611 procedures (5.1%), with a mean of 218 ±178 operations per year. Moreover, there was a trend for increase of overall procedures (r²=0.879) over the decade, equally observed in both open (r²=0.727) and laparoscopic (r²=0.887) interventions. When comparing the total growth of appendectomies over the years, the rise of laparoscopic surgery was 13,260% (median of 21.6% - IQR: 15.0% - 59.6%), while the traditional open intervention was 59.0% (median of 5.7% - IQR: 0.3% - 8.4%) (Mann-Whitney U=18.50; p<0,05). 

**Table 1:** Number of procedures and deaths related to the appendectomies performed in the Unified Health System, state of Bahia (2008 - 2019).

	Number of admissions per procedure			Number of deaths per procedure		
	Overall appendectomy	Open appendectomy	Laparoscopic appendectomy	Overall appendectomy	Open appendectomy	Laparoscopic appendectomy
2008	3.215	3.210	5	14	14	0
2009	3.549	3.538	11	21	21	0
2010	3.750	3.737	13	28	28	0
2011	3.832	3.711	121	32	32	0
2012	4.188	4.007	181	24	24	0
2013	4.403	4.226	177	15	14	1
2014	4.143	3.929	214	16	16	0
2015	4.673	4.384	289	24	24	0
2016	4.988	4.665	323	18	18	0
2017	5.016	4.690	326	18	16	2
2018	5.495	5.101	394	22	22	0
2019	5.772	5.104	668	18	18	0
Total	53,024	50,302	2,722	250	247	3
Mean	4,418.67	4,191.83	226.83	20.83	20.58	0.25
Standart deviation	756.2	584.6	183.3	5.2	5.4	0.6
Median	4,295.5	4,116.5	197.5	19.5	19.5	0
IQR (Q1-Q3)	3,812 - 4,995	3,731 - 4,671	94 - 324	18 - 24	16 - 24	0 - 0
r²	0.867	0.700	0.876	0.0563	0.0466	0.0235
Forecast (2025)	6,908	6,112	797	17	16	1
Upper limit CI95%	7,134	6,298	907	42	43	2
Lower limit CI95%	6,683	5,926	687	0	0	0

Throughout this period, a total of 250 deaths were due to appendectomies, which was also higher among the open technique (247 deaths - 98.8%), recording a mean of 21 ± 5 deaths per year. Laparoscopic surgery was responsible for 3 (1.2%) deaths and a mean of 0.3 ± 0.6 deaths per year (Table 1). The general appendectomy-related mortality rate was 4.7/1,000 procedures; however, it was significantly higher among open surgery patients (4.9/1,000 procedures) when compared to laparoscopic patients (1.1/1,000 procedures) (p<0.05). More extensive analysis also revealed a higher risk of death associated with the traditional laparotomic appendectomy (RR=4.5; CI95%=1.4-14.05; ꭓ²=7.2; p<0.05). Using the mathematical model, the data forecasted that the overall mortality rate might be reduced to 1.44/1,000 procedures in 2025.

Analysis regarding the length of stay revealed a total of 218,987 days, with an overall mean of 4.19 ± 0.4 days per operation. Open appendectomy (17,642 days/year; SD=1,200) presented a higher mean of the total of days spent on hospitalization days per year compared to laparoscopy (607 days/year; SD=439.7) (Table 2). Patients who underwent laparotomic appendectomy presented a significantly longer median hospital stay - a median of 4.15 days - compared to laparoscopy - a median of 2.7 days (Mann-Whitney U=132.00; p<0.05). Open appendectomy showed a trend to increase the total number of hospitalization days (r²=0.741), with a relative growth rate of +1.6% (SD=0.06) per year. However, it was accompanied by a trend to shorten the median of in-hospital stay (in days) (r²=0.958), with an average reduction of -2.74 ± 0.021% per year. In concomitance with these results, the mathematical model for forecasting the mean of hospital stay per intervention (in days) predicted a reduction to 3.0 days for open surgery and 1.8 days for laparoscopic appendectomy in 2025.

**Table 2:** Hospital days and length of stay in hospital per procedure in the Unified Health System, state of Bahia (2008 - 2019).

	Total of hospital stay (in days)			Mean of hospital stay (in days) per admission		
	Overall appendectomy	Open appendectomy	Laparoscopic appendectomy	Overall appendectomy	Open appendectomy	Laparoscopic appendectomy
2008	15.871	15.860	11	4.9	4.9	2.2
2009	16.470	16.431	39	4.6	4.6	3.5
2010	17.002	16.928	74	4.5	4.5	5.7
2011	16.874	16.562	312	4.4	4.5	2.6
2012	18.042	17.536	506	4.3	4.4	2.8
2013	18.539	17.962	577	4.2	4.3	3.3
2014	16.911	16.316	595	4.1	4.2	2.8
2015	19.013	18.116	897	4.1	4.1	3.1
2016	19.993	19.108	885	4	4.1	2.7
2017	19.227	18.402	825	3.8	3.9	2.5
2018	20.982	19.959	1.023	3.8	3.9	2.6
2019	20.063	18.519	1.544	3.5	3.6	2.3
Total	218.987	211.699	7.288	-	-	-
Mean	18.249	17.642	607	-	-	-
Standart deviation	1.568	1.200	439.7	-	-	-
Median	18.290	17.749	586	4.1	4.2	2.7
IQR (Q1-Q3)	16.901.7 - 19.418.5	16.529.2 - 18.431.2	252.5 - 888	4 - 4.4	4.1 - 4.5	2.6 - 3.1
r²	0.773	0.741	0.036	0.958	0.954	0.0643
Forecast (2025)	23.100	21.092	1.912	2.9	3.0	1.8
Upper limit CI95%	24.158	22.223	2.130	3.2	3.5	4.9
Lower limit CI95%	22.042	19.961	1.695	2.6	2.6	0

Data from 2008 to 2019 regarding the annual total cost due to appendectomies in the state of Bahia was US$ 5,555,367.28, with an average expenditure of US$ 462,947.27 ± 98,153.88 per year, showing an increasing curve (r²=0.956), with a relative growth rate of +7.6 ± 0.08% per year (Table 3). The total cost per year with laparotomic appendectomy (median of US$ 435,663.02) was higher than the annual amount spent with laparoscopy (median of US$ 23,475.48) and this difference was statistically significant (Mann-Whitney U=144.0; p<0.05). Nevertheless, there was no difference related to the value spent per procedure performed between laparotomic (median of US$ 103.61) and laparoscopic (median of US$ 111.70) (Mann-Whitney U=102.0; p=0.088). 

**Table 3:** Total cost, mean cost of hospital admission and mean cost of hospital day due to appendectomy in the state of Bahia (2008 - 2019).

	Annual Total cost			Mean cost of Hospital admission		Mean cost of Inpatient day	
	Overall appendectomy	Open appendectomy	Laparoscopic appendectomy	Open appendectomy	Laparoscopic appendectomy	Open appendectomy	Laparoscopic appendectomy
2008	US$ 285,386.56	US$ 284,989.98	US$ 396.58	US$ 88.78	US$ 79.32	US$ 17.97	US$ 36.05
2009	US$ 361,811.46	US$ 360,771.18	US$ 1,040.28	US$ 101.97	US$ 94.57	US$ 21.96	US$ 26.67
2010	US$ 391,244.73	US$ 389,572.44	US$ 1,672.29	US$ 104.25	US$ 128.64	US$ 23.01	US$ 22.60
2011	US$ 385,784.87	US$ 375,087.03	US$ 10,697.84	US$ 101.07	US$ 88.41	US$ 22.65	US$ 34.29
2012	US$ 432,874.34	US$ 413,992.06	US$ 18,882.28	US$ 103.32	US$ 104.32	US$ 23.61	US$ 37.32
2013	US$ 471,126.77	US$ 450,715.62	US$ 20,411.15	US$ 106.65	US$ 115.32	US$ 25.09	US$ 35.37
2014	US$ 447,150.23	US$ 420,610.43	US$ 26,539.80	US$ 107.05	US$ 124.02	US$ 25.78	US$ 44.60
2015	US$ 503,814.27	US$ 466,619.57	US$ 37,194.70	US$ 106.44	US$ 128.70	US$ 25.76	US$ 41.47
2016	US$ 540,205.29	US$ 501,881.98	US$ 38,323.30	US$ 107.58	US$ 118.65	US$ 26.27	US$ 43.30
2017	US$ 530,776.07	US$ 492,035.99	US$ 38,740.08	US$ 104.91	US$ 118.83	US$ 26.74	US$ 46.96
2018	US$ 584,426.94	US$ 537,099.95	US$ 47,326.99	US$ 105.29	US$ 120.12	US$ 26.91	US$ 46.26
2019	US$ 620,765.77	US$ 540,923.37	US$ 79,842.40	US$ 105.98	US$ 119.52	US$ 29.21	US$ 51.71
Total	US$ 5,555,367.28	US$ 5,234,299.61	US$ 321,067.67	-	-	-	-
Mean	US$ 462,947.27	US$ 436,191.63	US$ 26,755.64	US$ 103.61	US$ 111.70	US$ 24.58	US$ 38.88
Standart deviation	98,153.88	76,820.18	23,302.73	5.09	16.27	2.94	8.54
Median	US$ 459,138.50	US$ 435,663.02	US$ 23,475.48	US$ 105.10	US$ 118.74	US$ 25.43	US$ 39.39
IQR (Q1-Q3)	456,141.43 - 551,260.70	431,899.88 - 510,686.48	23,432.29 - 40,886.80	104.59 - 106.75	114.41 - 121.09	24.96 - 26.78	38.49 - 46.44
r²	0.956	0.925	0.0463	0.434	0.362	0.874	0.723
Forecast (2025)	US$ 773,313.72	US$ 670,638.02	US$ 176,394.42	US$ 111.64	US$ 143.55	US$ 33.69	US$ 63.64
Upper limit CI95%	US$ 795,645.76	US$ 710,765.56	US$ 271,169.33	US$ 129.46	US$ 176.39	US$ 38.76	US$ 80.75
Lower limit CI95%	US$ 750,981.69	US$ 630,510.48	US$ 81,619.52	US$ 93.82	US$ 110.71	US$ 28.61	US$ 46.52

When comparing costs with hospital (H) and professionals (P) services, open operation total costs (H: US$ 3,507,460.22 and P: US$ 1,726,839.39) were higher than laparoscopy total costs (H: US$ 221,530.11 and P: US$ 99,537.56), p<0.05 and p<0.05, respectively (Table 4). Furthermore, despite the significant difference between the median of professional costs per procedure (Mann-Whitney U=9.0; p<0.05), comparing laparotomic (median of US$ 34.60) and laparoscopic (median of US$ 35.99) techniques, there was no difference identified regarding the hospital service costs per surgical intervention (p=0.08), with medians of US$ 71.21 and US$ 80.55 per procedure, respectively.

**Table 4:** Hospital and professional services costs due to appendectomy in the state of Bahia (2008 - 2019).

	Cost with hospital services		Cost with professsional services	
	Open Appendectomy	Laparoscopic appendectomy	Open Appendectomy	Laparoscopic appendectomy
2008	US$ 182,502.22	US$ 225.01	US$ 102,487.76	US$ 171.57
2009	US$ 236,035.76	US$ 645.26	US$ 124,735.42	US$ 395.02
2010	US$ 256,948.68	US$ 1,029.69	US$ 132,623.76	US$ 642.60
2011	US$ 246,955.90	US$ 6,412.34	US$ 128,131.12	US$ 4,285.50
2012	US$ 274,336.79	US$ 12,456.17	US$ 139,655.27	US$ 6,426.11
2013	US$ 303,378.18	US$ 13,942.61	US$ 147,337.43	US$ 6,468.54
2014	US$ 283,100.04	US$ 18,228.99	US$ 137,510.39	US$ 8,310.81
2015	US$ 314,575.38	US$ 25,847.43	US$ 152,044.20	US$ 11,347.27
2016	US$ 340,890.85	US$ 26,449.41	US$ 160,991.14	US$ 11,873.89
2017	US$ 331,823.10	US$ 26,984.26	US$ 160,212.89	US$ 11,755.82
2018	US$ 365,584.04	US$ 33,186.67	US$ 171,515.92	US$ 14,140.32
2019	US$ 371,329.28	US$ 56,122.27	US$ 169,594.09	US$ 23,720.13
Total	US$ 3,507,460.22	US$ 221,530.11	US$ 1,726,839.39	US$ 99,537.56
Mean	US$ 292,288.35	US$ 18,460.84	US$ 143,903.28	US$ 8,294.80
Standart deviation	54,077.55	15,763.19	19,527.96	6,557.87
Median	US$ 293,239.11	US$ 16,085.80	US$ 143,496.35	US$ 7,389.68
Iqr (q1-q3)	254,450.49 - 334,090.04	5,066.68 - 26,583.12	131,500.60 - 160,407.45	3,374.77 - 11,785.34
Median cost per procedure	US$ 71.21	US$ 80.55	US$ 34.60	US$ 35.99
IQR (Q1-Q3)	68.03 - 71.85	66.28 - 84.07	34.03 - 34.90	35.51 - 37.28
r²	0.931	0.154	0.9038	0.2735
Forecast (2025)	US$ 465,900.27	US$ 124,635.75	US$ 202,220.27	US$ 28,698.99
Upper limit CI95%	US$ 492,473.80	US$ 191,391.12	US$ 220,547.33	US$ 32,418.72
Lower limit CI95%	US$ 439,326.75	US$ 57,880.38	US$ 183,893.21	US$ 24,979.26

## DISCUSSION

Acute appendicitis is the most common abdominal emergency requiring surgical intervention^6^ ^,^ ^10^. It must be considered for any individual presenting non-traumatic abdominal pain (acute abdomen), and if appendicitis is not the first diagnostic hypothesis, it surely should be the second one^7^ ^,^ ^12^. For this reason, reference services must have well-trained surgeons in both approaches, the laparotomic and minimally invasive procedures^4^ ^,^ ^10^ ^,^ ^11^. Even with this postulate, investigations and criticisms regarding the appendectomies practiced in the state of Bahia are essentially relevant since previous reports evinced that yet only a few public hospitals routinely perform the video-surgery technique in Salvador, the capital city of the state^6^.

Despite this reality in Bahia, the literature is consistent about the benefits of laparoscopic appendectomy. Biondi and colleagues (2016), when evaluating 593 patients from Italy, revealed shorter hospital stay, less need for analgesic medication, fewer complications, and lower incidence of wound infections associated with laparoscopy when compared to open surgery^7^. Minutolo et al (2014) investigated 230 patients undergoing appendectomies, comparing laparoscopic versus laparotomic operations, and showed a significantly lower prevalence of postoperative complications (2.9% versus 13.2%) and shorter length of hospital stay (2.75 days versus 3.87 days) associated with laparoscopy^11^. An observational retrospective study (2011) in the United States of America (USA) assessed 29,802 cases, comparing the minimally invasive operation to the open procedure, and demonstrated less overall morbidity (4.2% versus 6.91%), less need for intensive care unit admission (2.04% versus 3.68%), shorter length of stay (1.74 days versus 2.45 days), fewer 30-day readmissions (1.86% versus 2.97%) and lower overall mortality (0.07% versus 0.17%) associated with the video-surgery^13^. 

In our analyses, patients from Bahia undergoing laparoscopic appendectomy showed lower mortality rate (1.1/1,000 procedures) and shorter length of hospital stay (median of 2.7 days) compared to open technique (4.9/1,000 procedures and median of 4.15 days, respectively), a technique that also offered a higher risk of death to patients (RR=4.5; p<0.05). Similar results were also observed by Santos et al. (2017) when analyzing 684,278 appendectomies performed in Brazil in the years 2008-2014, which also linked a lower mean of hospital stay in days (3.6 days versus 3.8 days) and lower mortality rate (0.12% versus 0.28%) to the laparoscopic intervention^10^. 

On the other hand, conflicting findings were presented by Katkhouda et al. (2005) in a 247-patient study in the USA. Unlike other experiences, there were no significant differences between postoperative complications, scores on pain scale, length of stay, regardless of the technique, and, contradictorily, laparoscopy presented earlier complications requiring reoperation and longer operating time^14^.

In our findings, the increase in the total number of hospitalizations was accompanied by a progressively shorter median length of stay per procedure for both open and laparoscopic techniques from 2008 (4.6 days and 3.5 days) to 2019 (3.6 days and 2.3 days), with notorious emphasis on the minimally invasive procedure (p<0.05). This shortening of the length of stay might suggest earlier discharges over the studied years, which, in turn, can reflect in a progressively greater supply of hospital beds - in a state that chronically suffers from a scarcity of available hospital beds. These positive observations are corroborated by our forecast for the length of hospital stay per procedure in 2025, which predicts a decrease to 3.0 days (for open surgery) and 1.8 days (for video surgery). It is important to highlight that the surgical aggressiveness of an open procedure also contributes to the intrinsic difference in the length of stay between techniques.

Another important rising question is the applicability of laparoscopic surgery for complicated cases. A complicated case is defined as acute appendicitis that evolved with perforation or intraabdominal abscess, and the clinical benefit of laparoscopy for these patients is still questionable for some authors^8^ ^,^ ^13^. Tiwari et al. (2005) critically analyzed 10,535 complicated acute appendicitis patients, and demonstrated less overall morbidity, less need for intensive care unit admission, shorter length of stay, fewer 30-day readmissions, lower costs, and lower overall mortality correlated with the laparoscopic appendectomy^13^. Ball et al. (2004) obtained similar conclusions with 95 complicated cases of acute appendicitis, associating shorter length of stay, fewer wound infections, intraabdominal abscess, and hematoma with the minimally invasive appendectomy^8^, which reinforces the idea that this technique may also be safe and effective for complicated appendicitis. Although this assessment is important, it was not possible to be performed in our study since our database does not discriminate the type of operations related to the complication status of the registered patient, representing an important limitation of the present investigation.

Our data showed a significant rise in the total number of appendectomies performed in the state of Bahia in the years 2008-2019, for both open (total of 59.0%) and laparoscopic (total of 13,260.0%) modalities. Further, overall survival has improved for both techniques in the past years, but it was greater in the laparoscopic appendectomies group (r²=0.875), with an annual mean increase of 106.9% (SD=2.31), which might represent an overall improvement in the learning curve of surgeons in Bahia. Comparable data were also observed in the USA since the frequency of video surgery for appendicitis increased from 20.6% in 1998 to 70.8% in 2008 in Pennsylvania ^15^. Furthermore, Santos et al. (2017) identified similar growth in the performance of laparotomic and laparoscopic operations in Brazil between 2008 and 2014, with a total increase of 25.0% and 279.7%, respectively^10^. 

In contrary to these findings, there was an important reduction of 1,938 hospital beds offered by the Unified Health System (SUS) in Bahia between 2006 and 2015. In these years, the East and South macroregions of the state (mostly represented by Salvador and Porto Seguro, respectively) had a reduction of 453 and 266 surgical beds, respectively, and the Southwest macroregion (mostly represented by Vitória da Conquista) had an increase of 168 surgical beds, which, in overall, represented a reduction of 468 surgical beds in the state^16^. In parallel, it is noteworthy that the Unified Health System presents a structural branch of private/public partnership, in which private beds are supplied for public use on a complementary basis^16^. From this perspective, the complementary health system in Bahia provided an increase of 68.3% of hospital beds in the SUS^16^, which leads us to infer that this may have contributed, in turn, to the rise of appendectomies during the period of our analysis.

The proportion of open/minimally invasive surgery is considerably discrepant in our data, compared to other studies with large populations. A metanalysis comprising 25 randomized controlled trials involving 4,694 patients, between 1992 and 2010, reported 2,220 laparoscopic and 2,474 laparotomic procedures for acute appendicitis - a proportion of 1:1.11^17^. Moreover, Ingraham et al. used the American College of Surgeons/National Surgical Quality Improvement Program database to perform a multicenter analysis encompassing 222 hospitals and 32,683 patients. The authors revealed that laparoscopy (n=24,969) outnumbered open appendectomy (n=7,714)^9^. These findings suggest that the state of Bahia may need to enhance the number of educational centers for surgeons stimulating training in laparoscopic procedures and to improve the required infrastructure for video surgery at the reference hospitals, in the state. 

In the current interconnected world, the rational use of available resources, especially regarding budget or funding services, is the main focus of many administrative strategies. This is not different in the Brazilian Unified Health System, which embraces health as a constitutional right of all citizens and a duty of the State. In Bahia, the total number of open procedures outnumbered the laparoscopic procedures, therefore the total cost with laparotomy was higher, as expected. However, our financial analyses did not show a significant difference between the median cost per procedure regarding the laparotomy and laparoscopy (medians of US$ 103.61 versus US$ 111.70; p=0.08). Our results are in agreement with studies by Minutolo et al.^11^ (means of 2,337 € versus 2,282 €; p=0.81), Nguyen et al.^18^ (means of US$ 6,260 versus US$ 6,242; p=0.7), Santos et al.^10^ in Brazil (means of R$ 537.88 versus R$ 500.06, not significant), and Wei et al.^17^ in a metanalysis (p=0.47). Parallelly, Tiwari et al. observed that video surgery (US$ 12,125) was significantly less expensive than open surgery (US$ 17,597) among patients with complicated appendicitis (p<0.01)^13^. Furthermore, our study demonstrated no significant differences in hospital service costs between laparotomic and laparoscopic interventions (p=0.08), with medians of US$ 71.21 and US$ 80.55 per procedure, respectively. Similarly, Minutolo et al., by investigating associations in means of hospital costs, showed no differences between laparoscopy (2,282 €) and traditional open surgery (2,337 €) (p=0.812)^11^.

Our findings have been challenged by other authors, such as Biondi et al.^7^, who observed higher mean costs per procedure (mean difference of 150.00 €), and higher costs with hospital services (mean difference of 1,195.00 €), associated with laparoscopic appendectomy, which might represent an obstacle to its greater use. In Bahia, our analysis on professional costs per appendectomy demonstrated a significant difference between minimally invasive procedure (median of US$ 34.60) and laparotomy (median of US$ 35.99) (Mann-Whitney U=9.0; p<0.05). However, the median difference in professional costs was US$ 1.39 more expensive with laparoscopy, and the real impact of this difference in hospital administration might be questionable.

Most public health care centers and systems in the world face financial problems. The economic outcome of a public health system is not often related to the professional or work organization, but considerable budget problems resulting from underestimating the cost of surgical interventions funded by the public services^19^. In the collective thinking, laparoscopy is a type of intervention that has been over-expensive for public health services to be able to afford it routinely, but our findings showed no differences in the amounts paid per procedure or in the costs of hospital services, and registered a small difference regarding professional costs between the techniques. The literature suggests to the initial installation of video-surgery services is expensive, as well as staff training, and equipment purchases. On the other hand, laparoscopic procedures may impact on a greater long-term reduction in expenses since it has been repeatedly associated with lower rates of postoperative wound infections, early recovery, shorter hospitalization time, minimal demand for drugs in postoperative recovery, and a minimum number of postoperative complications in a vast number of conditions. All these benefits may, in turn, reduce overall treatment costs^19^. Several authors have demonstrated the same observations, with laparoscopic cholecystectomy^19^, laparoscopic colorectal cancer treatment^20^, and, as our investigation, laparoscopic appendectomy^11^ ^,^ ^17^ ^,^ ^18^.

Limitations of our study included its design and database (retrospective and observational study with secondary data from DATASUS), whose nature did not allow us to discriminate sex, age, comorbidities, or nutritional preconditions of patients. Moreover, it was not possible to evaluate the appendicitis status at admission, i.e., the severity of illness and complications, nor the surgical risk of patients, for instance, the American Society of Anesthesiology (ASA) Classification. Therefore, the present study could not correlate initial patient clinical status and worst outcomes regarding distinct techniques. Besides, it was not possible to observe the post-procedure evolution and determine which surgical technique achieved better outcomes on patients’ recovery. Since this is an ecological study, the database also does not provide information about human resources and hospital infrastructure for laparoscopic procedures between health services, neither training status in video-surgery of surgeons. Thus, we emphasize the need for multicenter and high-quality randomized controlled trial studies to further analyze this aspect, and to have more robust data in the state of Bahia.

## CONCLUSION

In conclusion, our results showed the advantages of the laparoscopic appendectomies over open surgery in public health services in the state of Bahia, including lower procedure-related mortality rate and shorter hospitalization time, with positive forecasts for 2025. Furthermore, this did not imply higher median costs associated with the minimally invasive procedure. Although the benefits related to laparoscopic appendectomies are demonstrated in our study, the number of these procedures performed in Bahia is still minimal, which, in turn, stimulates the expansion of public health services equipped with video surgery and more medical residency centers in general surgery with laparoscopic training. Nevertheless, it is important to emphasize that, in the light of current knowledge, we strongly recommend that the choice of the technique should be based on the doctor-patient communication and the surgeon’s experience and preference.

## References

[1] Lima AP, Vieira FJ, Oliveira GPDM, Ramos PDS, Avelino ME, Prado FG (2016). Clinical-epidemiological profile of acute appendicitis retrospective analysis of 638 cases. Rev. Col. Bras. Cir.

[2] Ferris M, Quan S, Kaplan BS, Molodecky N, Ball CG, Chernoff GW (2017). The Global Incidence of Appendicitis A Systematic Review of Population-based Studies. Ann Surg.

[3] Abdelazeem M, Elnaiem H, AAZ B, Alanazi OA, AIA R, ARH S (2020). Acute Appendicitis Neglected to Rupture Review. Article. eIJPPR.

[4] Ceresoli M, Zucchi A, Allievi N, Harbi A, Pisano M, Montori G (2016). Acute appendicitis Epidemiology, treatment and outcomes- analysis of 16544 consecutive cases. World J Gastrointest Surg.

[5] BRASIL Sistema de Informações em Morbidade Hospitalar - Departamento de Informática do Sistema Único de Saúde [Internet].

[6] Maia G (2016). Análise da apendicectomia videolaparoscópica realizada em hospital público de referência em Salvador, Bahia (Brasil) [Internet].

[7] Biondi A, Di Stefano C, Ferrara F, Bellia A, Vacante M, Piazza L (2016). Laparoscopic versus open appendectomy A retrospective cohort study assessing outcomes and cost-effectiveness. World J Emerg Surg [Internet].

[8] Ball CG, Kortbeek JB, Kirkpatrick AW, Mitchell P (2004). Laparoscopic appendectomy for complicated appendicitis An evaluation of postoperative factors. Surg Endosc Other Interv Tech.

[9] Ingraham AM, Cohen ME, Bilimoria KY, Pritts TA, Ko CY, Esposito TJ (2010). Comparison of outcomes after laparoscopic versus open appendectomy for acute appendicitis at 222 ACS NSQIP hospitals. Surgery.

[10] Dos Ssantos F, Cavasana GF, De Campos T (2017). Perfil das apendicectomias realizadas no Sistema Público de Saúde do Brasil Rev. Col. Bras. Cir.

[11] Minutolo V, Licciardello A, Di Stefano B, Arena M, Arena G, Antonacci V (2014). Outcomes and cost analysis of laparoscopic versus open appendectomy for treatment of acute appendicitis 4-years experience in a district hospital. BMC Surg.

[12] Townsend C, Beauchamp D, Evers M, Mattox K (2016). Sabiston Textbook of Surgery - The Biological Basis of Modern Surgical Practice.

[13] Tiwari MM, Reynoso JF, Tsang AW, Oleynikov D (2011). Comparison of outcomes of laparoscopic and open appendectomy in management of uncomplicated and complicated appendicitis. Ann Surg.

[14] Katkhouda N, Mason RJ, Towfigh S, Gevorgyan A, Essani R, Barbul A (2005). Laparoscopic versus open appendectomy A prospective randomized double-blind study. In: Annals of SurgeryAnn Surg.

[15] McGrath B, Buckius MT, Grim R, Bell T, Ahuja V (2011). Economics of appendicitis Cost trend analysis of laparoscopic versus open appendectomy from 1998 to 2008. J Surg Res.

[16] Campello IC de S (2016). Distribuição dos leitos hospitalares na Bahia, Brasil, entre 2006 e 2015 segundo o CNES [Internet].

[17] Wei B, Qi CL, Chen TF, Zheng ZH, Huang JL, Hu BG (2011). Laparoscopic versus open appendectomy for acute appendicitis A metaanalysis. Surg Endosc.

[18] Nguyen NT, Zainabadi K, Mavandadi S, Paya M, Stevens CM, Root J (2004). Trends in utilization and outcomes of laparoscopic versus open appendectomy. Am J Surg.

[19] Smigielskimigielski JA, Piskorz L, Koptas W (2015). Comparison of treatment costs of laparoscopic and open surgery. Wideochir Inne Tech MaloinwazyjneWideochirurgia I Inne Tech Maloinwazyjne.

[20] Gehrman J, Angenete E, Björholt I, Lesén E, Haglind E (2020). Cost-effectiveness analysis of laparoscopic and open surgery in routine Swedish care for colorectal cancer. Surg Endosc.

